# Quantitative Imaging of White and Gray Matter Remyelination in the Cuprizone Demyelination Model Using the Macromolecular Proton Fraction

**DOI:** 10.3390/cells8101204

**Published:** 2019-10-05

**Authors:** Marina Khodanovich, Anna Pishchelko, Valentina Glazacheva, Edgar Pan, Andrey Akulov, Mikhail Svetlik, Yana Tyumentseva, Tatyana Anan’ina, Vasily Yarnykh

**Affiliations:** 1 Laboratory of Neurobiology, Research Institute of Biology and Biophysics, Tomsk State University, Tomsk 634050, Russia; 2 Institute of Cytology and Genetics, Siberian Branch of the Russian Academy of Sciences, Novosibirsk 630090, Russia; 3 Department of Radiology, University of Washington, Seattle, WA 98109, USA

**Keywords:** macromolecular proton fraction, MPF, myelin, magnetic resonance imaging, cuprizone model, demyelination, remyelination, oligodendrocyte precursors, oligodendrocytes, immunohistochemistry

## Abstract

Macromolecular proton fraction (MPF) has been established as a quantitative clinically-targeted MRI myelin biomarker based on recent demyelination studies. This study aimed to assess the capability of MPF to quantify remyelination using the murine cuprizone-induced reversible demyelination model. MPF was measured in vivo using the fast single-point method in three animal groups (control, cuprizone-induced demyelination, and remyelination after cuprizone withdrawal) and compared to quantitative immunohistochemistry for myelin basic protein (MBP), myelinating oligodendrocytes (CNP-positive cells), and oligodendrocyte precursor cells (OPC, NG2-positive cells) in the corpus callosum, caudate putamen, hippocampus, and cortex. In the demyelination group, MPF, MBP-stained area, and oligodendrocyte count were significantly reduced, while OPC count was significantly increased as compared to both control and remyelination groups in all anatomic structures (*p* < 0.05). All variables were similar in the control and remyelination groups. MPF and MBP-stained area strongly correlated in each anatomic structure (Pearson’s correlation coefficients, r = 0.80–0.90, *p* < 0.001). MPF and MBP correlated positively with oligodendrocyte count (r = 0.70–0.84, *p* < 0.01 for MPF; r = 0.81–0.92, *p* < 0.001 for MBP) and negatively with OPC count (r = −0.69–−0.77, *p* < 0.01 for MPF; r = −0.72–−0.89, *p* < 0.01 for MBP). This study provides immunohistological validation of fast MPF mapping as a non-invasive tool for quantitative assessment of de- and remyelination in white and gray matter and indicates the feasibility of using MPF as a surrogate marker of reparative processes in demyelinating diseases.

## 1. Introduction

New therapies enabling regeneration of damaged myelin may offer potential for restoring neurological function in multiple sclerosis (MS) and other demyelinating diseases [1,2,3]. Quantitative imaging biomarkers of remyelination are of critical importance for the development and clinical testing of myelin repair therapies [4,5]. Partial remyelination associated with the recruitment and proliferation of oligodendrocyte precursor cells (OPC) is a known phenomenon in MS lesions, which, however, usually fails to completely restore damaged brain tissue [1,6,7]. Although significant efforts were focused on identifying magnetic resonance imaging (MRI) signatures of remyelination in MS lesions [4,8,9], no existing technique provides sufficient sensitivity and specificity to myelin to be used as a routine clinical tool for remyelination monitoring [4]. Furthermore, the lesions detectable by MRI are known to represent only a small part of MS pathology, which is characterized by widespread microscopic demyelination of white and gray matter [10,11,12]. Assessment of remyelination in normal-appearing brain tissues remains unachievable by using any clinical imaging method to date.

A new MRI method, fast macromolecular proton fraction (MPF) mapping [12,13,14,15,16,17,18,19,20], has demonstrated promise as a reliable clinical and preclinical tool for quantitative imaging of demyelination [12,14,15,16,17,18] and myelin development [19,20]. This method is based on the magnetization transfer (MT) effect and enables quantification of the number of macromolecular protons that are involved into cross-relaxations with water protons [13]. In recent clinical studies, MPF mapping provided the capability to quantify microscopic demyelination in both white and gray matter caused by MS [12,15] and mild traumatic brain injury [16]. MPF mapping has been extensively validated by histology in animal models including the normal brain and C6 glioma in rats [21], cuprizone-induced demyelination in mice [17], and ischemic stroke in rats [18], where it demonstrated strong correlations with the myelin content across white and gray matter anatomic structures and lesions. However, to the best of our knowledge, this method has not been tested in the setting of remyelination.

Cuprizone-induced toxic demyelination in mice is a common animal model of MS [22,23,24,25,26], which is frequently used in preclinical studies of remyelination therapies [5,23,24,25]. In this model, demyelination is induced by the administration of the copper chelator cuprizone, causing selective oligodendrocyte apoptosis followed by extensive demyelination of both white and gray matter [22,23,24,25,26]. If cuprizone treatment is discontinued within a certain timeframe, spontaneous remyelination and functional recovery (complete or incomplete depending on treatment regimen) typically occur [22,23,24,25,26]. Although cuprizone intoxication is considered a reductionist model of MS devoid of the autoimmune inflammatory component, it reproduces certain pathological features of human disease including diffuse demyelination of white and gray matter, microglial activation, astrogliosis, axonal damage, and remyelination associated with OPC proliferation and oligodendrocyte repopulation [22,23,24,25,26]. The cuprizone model also offers substantial convenience for the evaluation of quantitative imaging methods targeted at the assessment of normal-appearing brain tissues, because cuprizone induces demyelination that affects the whole brain at the microscopic level without the formation of focal lesions, and appears to a variable extent across anatomic structures [17,24,25,26].

The primary objective of this study was to assess the capability of fast MPF mapping to quantify remyelination in white and gray matter using the cuprizone model. Additionally, we sought to investigate a relationship between MPF and markers of oligodendrogenesis, such as the number of oligodendrocytes and OPC, which are frequently used as outcome measures in preclinical studies of myelin repair therapies.

## 2. Materials and Methods

### 2.1. Animals and Experimental Design

All animal experiments were performed in accordance with the rules adopted by the European Convention for the Protection of Vertebrate Animals used for Experimental and Other Scientific Purposes. The experimental protocol was approved by the Bioethical Committee of the Institute of Cytology and Genetics of the Siberian Branch of the Russian Academy of Sciences (Protocol number 25) and the Bioethical Committee of the Biological Institute at Tomsk State University (Protocol number 3, Registration No. 8). Eight-week-old CD1 male mice were obtained from the vivarium of the E.D. Goldberg Institute of Pharmacology and Regenerative Medicine of the Siberian Branch of the Russian Academy of Sciences (Tomsk, Russian Federation). Animals were housed with a 12-h dark-light cycle at a temperature of 21 ± 2 °C, and humidity of 40 ± 2%. Food and water were provided ad libitum.

After 10 days of quarantine, the animals were divided into three groups: the control group (n = 4), the demyelination group (n = 4), and the remyelination group (n = 5). Mice in the demyelination group received 0.5% cuprizone (Bis(cyclohexanone)oxaldihydrazone, Sigma-Aldrich, St. Louis, MO, USA) with standard chow for 10 weeks. The remyelination group returned to a normal diet after 5 weeks of cuprizone treatment. The controls received standard chow for 10 weeks. After 10 weeks of cuprizone treatment, the mice were MRI-scanned with an MPF mapping protocol. Imaging was performed under isoflurane anesthesia (1.5–2% in oxygen) with respiratory monitoring during the scan. The mice were then transcardially perfused with 4% paraformaldehyde (PFA) under ether anesthesia. Brains were removed and fixed overnight in PFA at 4 °C. The brains were then cryoprotected in a graded concentration of sucrose in phosphate buffer (24 h at 10% and 24 h at 20%) at 4 °C, frozen in liquid nitrogen, and stored at −80 °C prior to immunohistochemical staining.

### 2.2. MRI Acquisition and Processing

The mice were imaged on an 11.7 Tesla horizontal-bore animal MRI scanner (BioSpec 117/16 USR; Bruker BioSpin, Ettlingen, Germany) with the manufacturer’s four-channel mouse brain surface coil. A fast high-resolution single-point 3D MPF mapping protocol was implemented as described previously [17]. The protocol included the following sequences applied in the coronal plane with a 3D field-of-view of 20 × 20 × 24 mm:

(1) MT-weighted spoiled gradient echo (GRE): repetition time (TR) = 22 ms, echo time (TE) = 2.5 ms, flip angle (FA) = 9°, spectral bandwidth (BW) = 125 kHz, off-resonance saturation by the Gaussian pulse with an offset frequency of 4500 kHz, effective FA = 900°, a duration of 10 ms, 3D matrix 200 × 200 × 48, spatial resolution 100 × 100 × 500 µm^3^, four signal acquisitions, and a scan time of 10 min 34 s;

(2) T_1_-weighted spoiled GRE: TR = 16 ms, TE = 2.5 ms, FA = 16°, BW = 125 kHz, 3D matrix 200 × 200 × 48, spatial resolution 100 × 100 × 500 µm^3^, four signal acquisitions, and a scan time of 7 min 41 s;

(3) Proton-density (PD)-weighted spoiled GRE: TR = 16 ms, TE = 2.5 ms, FA = 3°, BW = 125 kHz, 3D matrix 200 × 200 × 48, spatial resolution 100 × 100 × 500 µm^3^, four signal acquisitions, and a scan time of 7 min 41 s;

(4) B_0_ mapping using the dual-TE phase-difference method: TR = 20 ms, TE1 = 2.4 ms, TE2 = 4.1 ms, FA = 8°, BW = 200 kHz, 3D matrix 100 × 100 × 48, spatial resolution 200 × 200 × 500 µm^3^, one signal acquisition, and a scan time of 1 min 36 s;

(5) B_1_ mapping using the dual-TR actual flip-angle imaging (AFI) method: TR1 = 13 ms, TR2 = 65 ms, TE = 3.7 ms, FA = 60°, BW = 59.5 kHz, 3D matrix 100 × 100 × 48, spatial resolution 200 × 200 × 500 µm^3^, one signal acquisition, and a scan time of 4 min 45 s.

The GRE and AFI sequences were implemented with optimal radiofrequency and gradient spoiling based on the excitation pulse phase increments of 169° for GRE and 39° for AFI [27]. In all sequences, linear phase-encoding order with fractional (75%) k-space acquisition in the slab selection direction was used. The total scan time was about 35 min.

Reconstruction of MPF maps was carried out using custom-written C-language software based on the single-point algorithm with synthetic reference image normalization and correction of B_0_ and B_1_ field non-uniformities, as detailed elsewhere [13,14,17].

### 2.3. Immunohistochemistry

Coronal brain sections with 10 µm thickness were prepared using an HM525 cryostat (Thermo Fisher Scientific, Walldorf, Germany). Sections were obtained at two brain locations: −1.58 mm and +0.74 mm from bregma according to the mouse brain atlas [28].

Sections were stained using immunohistochemistry for myelin basic protein (MBP, the marker of myelin), 2′,3′-cyclic-nucleotide 3′-phosphodiesterase (CNP, the marker of myelinating oligodendrocytes), and neuro-glial antigen 2 (NG2, the marker of oligodendrocyte precursors). The primary antibodies were: goat polyclonal anti-MBP, (sc-13914, Santa Cruz Biotechnology, Dallas, TX, USA); mouse monoclonal anti-CNPase (Cat#MAB326, Merck Millipore, Burlington, MA, USA), rabbit polyclonal anti-NG2 (H-300), (sc-20162, Santa Cruz Biotechnology, USA), and goat polyclonal anti-DCX (C-18), (sc-8066, Santa Cruz Biotechnology, USA). The secondary antibody was donkey anti-goat AlexaFluor^®^ 488 (green color, code 705-545-147, Jackson ImmunoResearch, West Grove, PA, USA) or donkey anti-rabbit AlexaFluor^®^ 488 (green color, code 711-545-152, Jackson ImmunoResearch).

Slides were covered with mounting medium with DAPI (4′,6-diamidino-2-phenylindole, blue color, nuclear counter stain). Photography was performed using an Axio Imager Z2 microscope (Carl Zeiss, Oberkochen, Germany) and AxioVision 4.8 (Carl Zeiss) software with a MozaiX module, which enables reconstruction of the whole brain images by stitching a series of mosaic images. Additionally, 2′,3′-cyclic-nucleotide 3′-phosphodiesterase (CNP), neural/glial antigen 2 (NG2) stained sections were photographed using a confocal laser microscope LSM 780 NLO (Carl Zeiss).

### 2.4. Image Analysis

**Figure 1:**
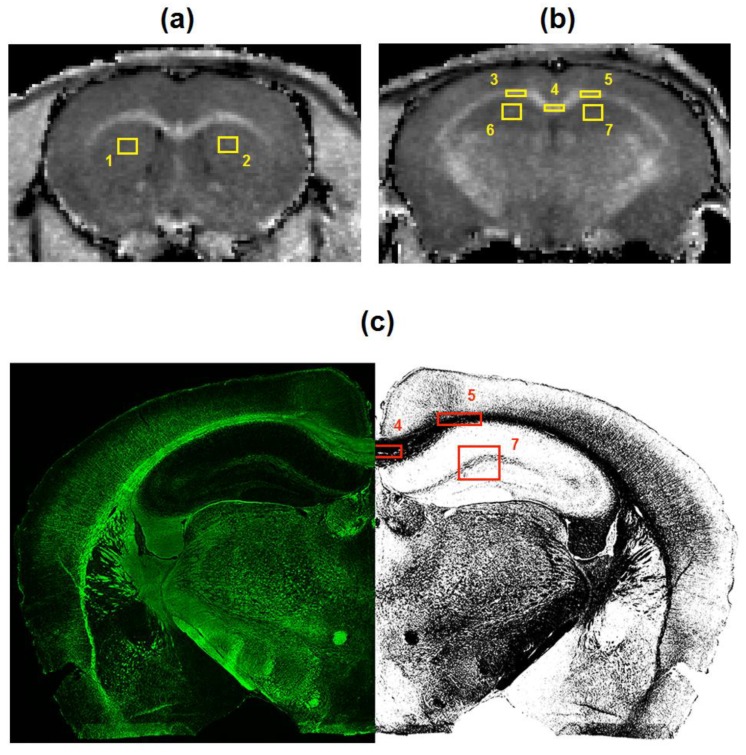
The scheme of image processing for macromolecular proton fraction (MPF) maps (**a,b**) and myelin basic protein (MBP)-stained images (**c**). Cross-sections of a 3D MPF map of a control mouse taken at the locations of +0.74 mm (a) and −1.58 mm (b) from bregma and superimposed with regions-of-interest (ROIs) corresponding to the following brain structures: 1, 2—caudate putamen; 3, 4, 5—corpus callosum; 6, 7—hippocampus, 8, 9, 10, 11—cortex. MBP-stained image at the location of −1.58 mm from bregma (c) is presented in the native (left) and binarized (right) forms with ROIs corresponding to the corpus callosum (5,6), hippocampus (7), and cortex (10,11).

MPF maps and microphotographs of MBP-, CNP-, and NG2-stained brain sections were analyzed using freely available ImageJ software (National Institutes of Health, Bethesda, MD, USA). Two brain locations (−1.58 mm and +0.74 mm from bregma) defined according to the mouse brain atlas [28] were chosen for quantitative analysis. Quantitative imaging variables were assessed in the regions-of-interest (ROIs) of standard sizes placed within the following anatomic structures: the central and distal parts of the corpus callosum, center of the caudate putamen, cortex, and hippocampus. The scheme of image analysis is presented in Figure 1. MPF values were quantified as previously described [17]. Myelin density on the MBP microphotographs was quantified in the above structures by the Otsu thresholding method [29] implemented as a plugin for ImageJ software [30]. A percentage of the total area of detected objects in the binarized images (Figure 1) was used as a surrogate measure of MBP density [31]. Myelinating oligodendrocytes (CNP-positive cells) and OPC (NG2-positive cells) were counted manually in the similarly positioned ROIs.

### 2.5. Statistical Analysis

All statistical analyses were carried out using Statistica 10.0 for Windows (StatSoft Inc, Tulsa, OK, USA). Mean values and standard errors of the mean were calculated for each variable in each anatomical structure. Normality of the data within animal groups and residuals in regression analyses was assessed using the Shapiro-Wilk’s test. No significant deviations from the normal distribution were found, and therefore, parametric analyses were used. MPF values and quantitative histology data were compared between the control, demyelination, and remyelination groups using a two-way repeated measures analysis of variance (ANOVA) followed by post hoc least significant difference (LSD) tests for individual anatomical structures.

The effects of demyelination and remyelination on MPF and histological variables in each anatomic structure were characterized by the effect sizes (Cohen’s d) calculated as the ratios of the mean differences to the pooled standard deviation. The d values for demyelination were calculated from the differences between the control and demyelination groups. The d values for remyelination were calculated from the differences between the demyelination and remyelination groups. Pearson’s correlation coefficient (r) and linear regression analysis were used to determine associations between variables across anatomical structures and animals. Statistical significance was defined as a *p* value less than 0.05.

## 3. Results

### 3.1. Effects of Demyelination and Remyelination on MPF, MBP Density, and the Counts of Myelinating Oligodendrocytes and OPC

**Figure 2:**
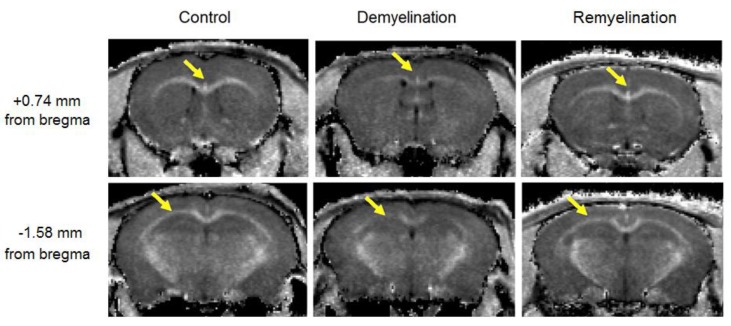
Example MPF maps of the mice from the control (left), demyelination (center), and remyelination (right) groups. Cross-sections of 3D MPF maps were taken through the caudate putamen (+0.74 mm from bregma, top) and hippocampus (−1.58 mm from bregma, bottom). Arrows show a visible reduction in MPF in the corpus callosum. MPF maps are presented with the grayscale range corresponding to 2–16% and window centered at 9%.

**Figure 3:**
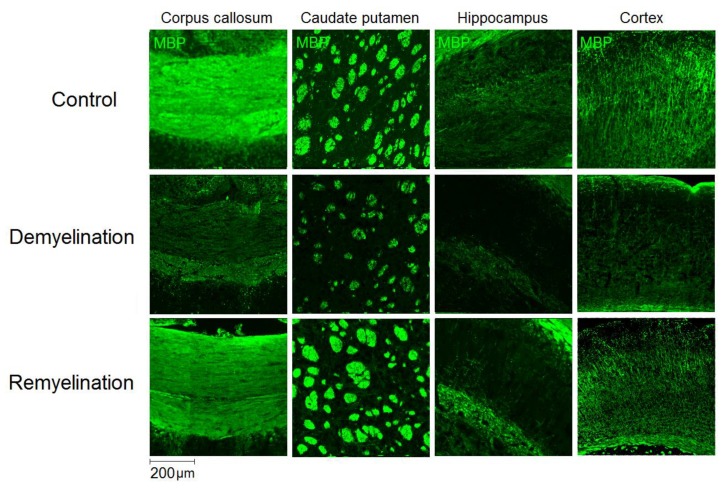
Effect of cuprizone-induced demyelination and remyelination after cuprizone discontinuation on MBP immunostaining. Example microphotographs of MBP-stained sections are presented for the corpus callosum, caudate putamen, cortex, and hippocampus with magnification of ×100.

**Figure 4:**
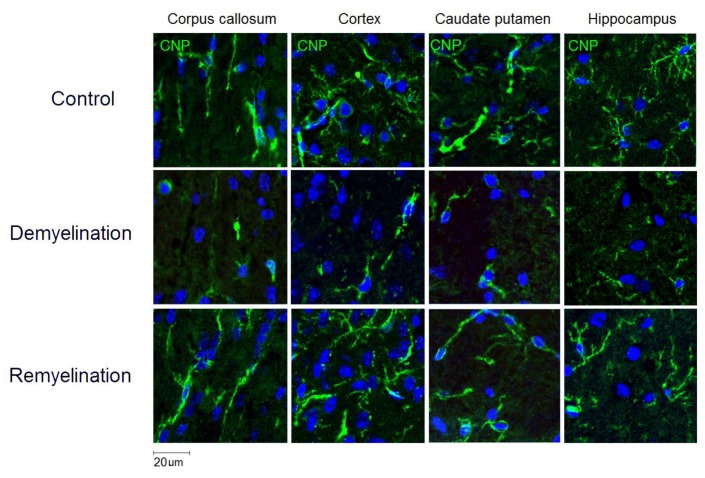
Effect of cuprizone-induced demyelination and remyelination after cuprizone discontinuation on the number of myelinating oligodendrocytes (CNP-positive cells). Example microphotographs of CNP-stained sections are presented for the corpus callosum, caudate putamen, cortex, and hippocampus with magnification of ×200.

**Figure 5:**
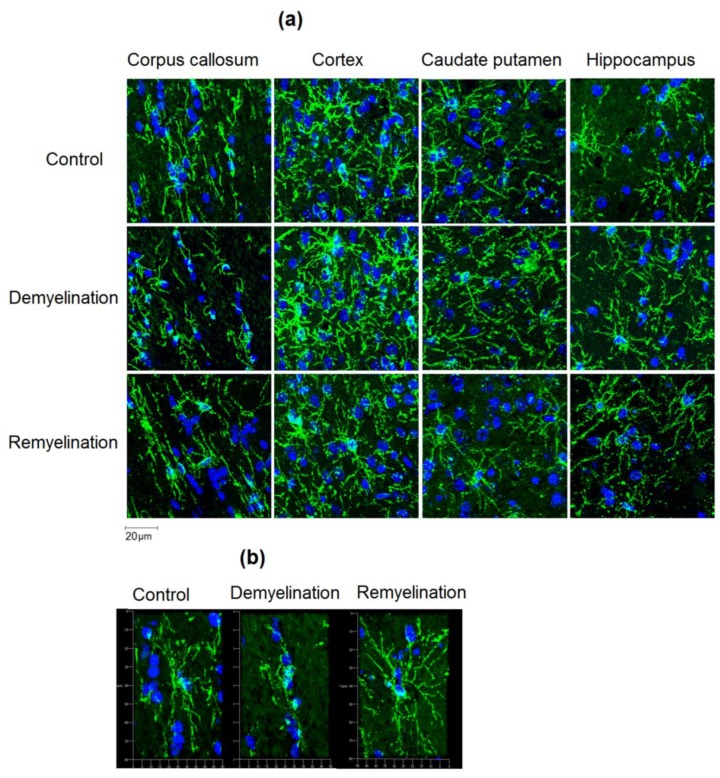
Effect of cuprizone-induced demyelination and remyelination after cuprizone discontinuation on the amount of OPC (NG2-positive cells). (**a**) Example microphotographs of NG2-stained sections corresponding to the corpus callosum, caudate putamen, cortex, and hippocampus with magnification of ×200. (**b**) 3D reconstructions of separate NG2-positive cells in the corpus callosum illustrating distinctions in OPC morphology. Magnification: ×200.

**Figure 6:**
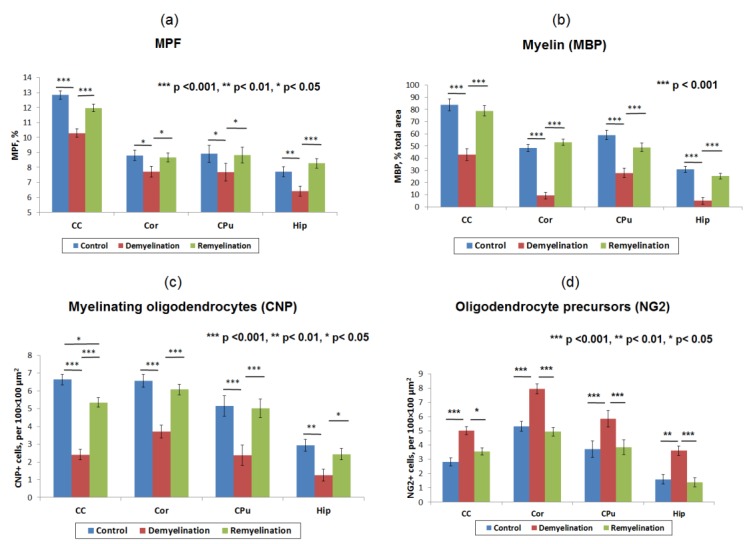
Summary statistics of MPF measurements and quantitative histological variables in the brain anatomic structures across the control, demyelination, and remyelination animal groups: MPF values (**a**), myelin content according to the percentage of MBP staining area (**b**), the count of myelinating oligodendrocytes (CNP-positive cells) (**c**), and OPC count (NG2-positive cells) (**d**). Anatomic structures are abbreviated as follows: CC—corpus callosum, Cor—cortex, CPu—caudate putamen, and Hip—hippocampus. Significant differences between the groups according to the LSD test are marked by asterisks: * – *p* < 0.05, ** – *p* < 0.01, and *** – *p* < 0.001. Error bars represent standard errors of the mean.

Example MPF maps of the murine brain from the control, demyelination, and remyelination groups at the two brain locations are demonstrated in Figure 2. Representative microphotographs corresponding to the investigated brain structures in the three animal groups taken from MBP-, CNP-, and NG2-stained sections are shown in Figure 3, Figure 4 and Figure 5, respectively. Summary statistics and group comparisons for MPF measurements and quantitative histological variables are presented in Figure 6. Cuprizone-treated mice demonstrated reduced contrast between white and gray matter on MPF maps as compared to control mice, particularly in the corpus callosum, whereas the maps from animals in the remyelination group showed visually normal tissue contrast (Figure 2). MPF values in both the corpus callosum and gray matter structures (caudate putamen, cortex, and hippocampus) were significantly smaller in the demyelination group as compared to the control and remyelination groups (Figure 6a). The control and remyelination groups did not show significant differences in MPF (Figure 6a). MBP immunostaining showed visible loss of myelin in all structures in the demyelination group and restoration of myelination patterns in the remyelination group (Figure 3). MBP quantitation by the percentage of the stained area showed a significant decrease in myelin density in all structures in the demyelination group relative to both control and remyelination groups and the absence of significant differences between the control and remyelination groups (Figure 6b). The count of myelinating oligodendrocytes showed a similar trend and was significantly reduced in the demyelination group as compared to the control and remyelination groups (Figure 4, Figure 6c). In the remyelination group, the CNP-positive cell count in the corpus callosum was significantly lower than that in controls, while no significant differences were found for other structures (Figure 6c). The amount of OPC showed an opposite trend, with a significant increase in the demyelination group relative to both control and demyelination groups in all brain structures (Figure 6d). There were no significant differences in the NG2-positive cell count between the control and remyelination groups. Another interesting observation is substantial changes in OPC morphology in the demyelination group, where these cells exhibited shorter and fewer processes or bipolar or star-like structures reflecting variable stages of immaturity. Notably, OPC in remyelinated animals showed highly branched morphology, similar to the control animals (Figure 5b).

Effect sizes characterizing de- and remyelination across the studied brain regions in a uniform dimensionless scale are summarized in Table 1. For all variables and anatomic regions, the effect sizes appeared very large (d > 1.3) according to the commonly used classification [32]. The d values tended to be larger in the absolute scale for MBP staining and CNP-positive cell count and comparable for MPF and NG2-positive cell count. For all variables except for the NG2-positive cell count, the effect sizes were fairly homogenous across the anatomic regions (Table 1).

### 3.2. Correlations between MPF and Quantitative Histology Variables

**Figure 7:**
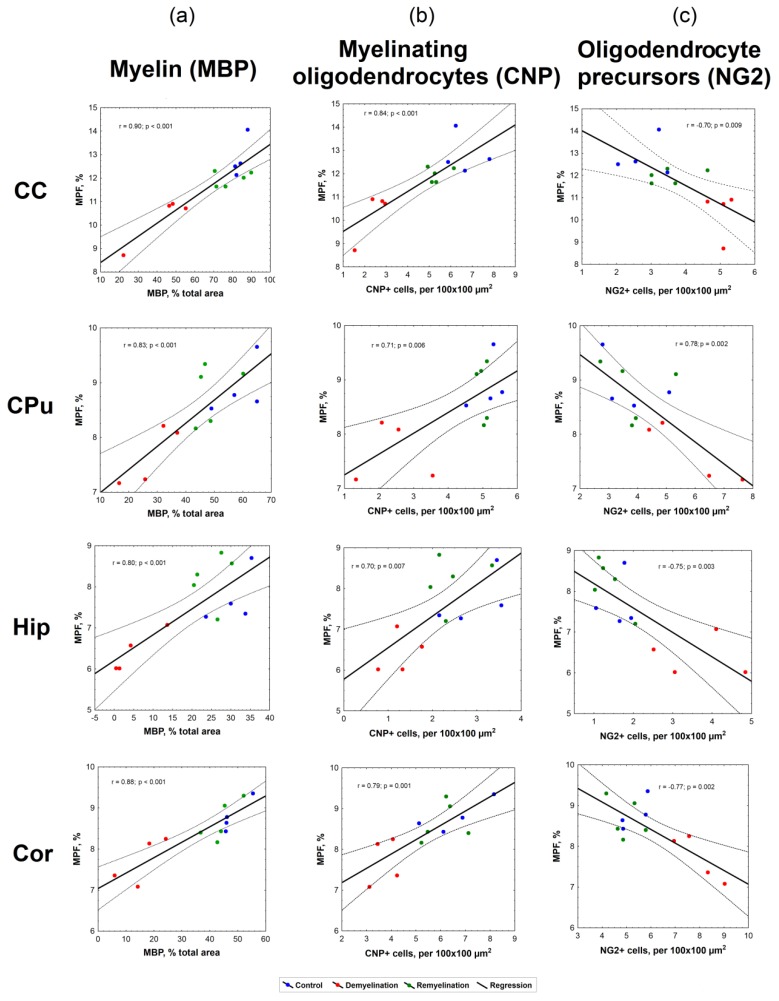
Linear regression analysis of MPF values vs. myelin content according to MBP staining (**a**), the number of myelinating oligodendrocytes according to CNP staining (**b**), and the number of OPC according to NG2 staining (**c**) in the corpus callosum, caudate putamen, hippocampus, and cortex. Blue, red and green dots correspond to the control, demyelination, and remyelination groups, respectively. Black lines depict the plots of the regression equations for the pooled datasets including all groups. Dotted lines represent confidence intervals of the regression lines.

The results of linear regression analyses for MPF values as a function of the myelin content, the number of myelinating oligodendrocytes and OPC are presented in Figure 7. The summary of regression analyses for all variables is provided in Table 2.

Correlations between all variables appeared statistically significant for each investigated brain anatomic structure (corpus callosum, caudate putamen, hippocampus, and cortex). MPF values strongly correlated with MBP-stained percentage of the total area (r = 0.80–0.90, *p* < 0.001), as seen in Figure 6a. Both MPF and MBP staining demonstrated strong positive correlations with the count of CNP-positive myelinating oligodendrocytes (Figure 6b, Table 2), although correlations for MPF (r = 0.70–0.84, *p* < 0.01) were slightly weaker than those for MBP (r = 0.81–0.92, *p* < 0.001). MPF and MBP also showed strong negative correlations with the amount of NG2-positive OPC (r = −0.69–−0.77, *p* < 0.01 for MPF; r = −0.72–−0.89, *p* < 0.01 for MBP), while the counts of CNP-positive and NG2-positive cells were negatively correlated in all anatomic structures (Figure 6c, Table 2).

## 4. Discussion

The results of this study demonstrate the capability of fast MPF mapping to accurately and reliably quantify remyelination in both white and gray matter. MPF showed very similar patterns of changes as compared to the immunohistochemical myelin marker MBP including a significant decrease caused by the cuprizone treatment and restoration to the nearly normal level after treatment discontinuation. These findings are further supported by strong correlations between MPF and MBP immunofluorescence in all investigated white and gray matter anatomic structures including the corpus callosum, caudate putamen, hippocampus, and cortex. It is important to emphasize that correlations between MPF and MBP in this study were assessed separately for each structure and, therefore, could not be driven by intrinsic distinctions in the myelin content between white and gray matter.

In the aspect of demyelination, our results confirm the conclusions of the previous study [17], which demonstrated close agreement between MPF and quantitative myelin histology in the assessment of cuprizone-induced myelin loss in white and gray matter across a series of anatomic structures. It should be pointed out that the earlier results [17] were achieved with luxol fast blue histological staining, whereas this study utilized immunohistochemistry with MBP. Similar findings obtained with different myelin markers provide extra confidence to the interpretation of MPF measurements in terms of the myelin content.

Cuprizone-induced demyelination has been extensively studied by various MRI methods as overviewed earlier [17]. At the same time, fewer MRI studies investigated remyelination in this model [33,34,35,36,37,38,39,40,41,42,43]. The majority of these publications [33,34,35,36,37,38,39,40] were limited to analyzing the behavior of imaging variables in the corpus callosum, which is known as the most susceptible to cuprizone-induced demyelination anatomic structure [22,23,24,25,26]. Several quantitative and non-quantitative imaging techniques, such as conventional T_1_- and T_2_-weighted imaging [34,43], T_2_ mapping [38,43], diffusion tensor imaging (DTI) [33,36,38,40,42,43], diffusion kurtosis imaging (DKI) [38,42], and magnetization transfer imaging in the traditional semi-quantitative [34,35,38,39,41] and quantitative [37] variants demonstrated significant trends in the corresponding imaging parameters consistent with de- and remyelination in the corpus callosum. Among the above studies, significant correlations of variable strength (absolute values of correlation coefficients in a range of 0.6–0.9) between myelin histology [35,37,39,40,41] or electron microscopy [34,38] and imaging variables in the corpus callosum were found for normalized T_1_- and T_2_-weighted signal intensities [34], T_2_ values [38], DTI-related indexes (mean diffusivity, radial diffusivity, and fractional anisotropy) [38,40], MT ratio (MTR, conventional semi-quantitative MT imaging index) [34,35,39,41], and pool size ratio (PSR) derived from quantitative MT measurements [37]. To the best of our knowledge, only four studies [33,41,42,43] investigated changes in MRI parameters associated with both de- and remyelination in regions other than corpus callosum anatomic regions, including a series of white matter fiber tracts [33,43], cortical gray matter [41,42,43], and subcortical gray matter structures (caudate putamen [41,43] and thalamus [43]). In some white matter regions outside the corpus callosum, T_2_ values showed significant differences associated with de- and remyelination [43], while the trends for diffusion tensor metrics were non-significant [33,43]. In the deep gray matter structures, the significant effects of de- and remyelination were found for T_2_ in the caudate putamen and thalamus [43] and for MTR in the caudate putamen [41]. MTR was also weakly (r = 0.46) but significantly correlated with histologically measured myelin content in the caudate putamen [41]. In the cortex, quantitative changes in T_2_ were significant for demyelination but failed to detect remyelination [43]. No significant effects of cortical demyelination or remyelination were identified for MTR [41]. DKI was the only quantitative MRI technique that was able to detect significant cortical changes consistent with cuprizone-induced demyelination and subsequent recovery [42]. However, DKI indexes in white and gray matter were reported to change in opposite directions [42], thus suggesting that the observed DKI parameter alterations were driven by factors other than myelination. None of earlier cuprizone model studies has reported quantitative correlations between histological myelin measures and imaging variables in the cortex and hippocampus. As evidenced by this and previous studies, only the fast MPF mapping method enables reliable in vivo quantitation of both demyelination and remyelination in a variety of white and gray matter structures, being in close agreement with histology.

Our observations in the investigated anatomic structures are in overall agreement with earlier immunopathological studies, which reported prominent cuprizone-induced myelin loss and oligodendrocyte depletion in the corpus callosum [22,23,24,25,26,40,44,45,46,47,48], cortex [45,46,47,48], caudate putamen [41,48,49], and hippocampus [45,48,50,51]. Similar to previous publications, we also observed remyelination accompanied with recovery of oligodendrocyte population in the corpus callosum [22,23,24,25,26,40,44,45,46,47], cortex [45,46,47], caudate putamen [41], and hippocampus [45] after cuprizone discontinuation. These processes were paralleled by an increased OPC count in all structures during the demyelination phase followed by its restoration to a nearly normal level during the remyelination phase. Similar patterns of OPC population changes were reported for the corpus callosum [40,44,45], cortex [45,47], and hippocampus [45], though some studies did not find significant OPC proliferation in the basal ganglia [49] and hippocampus [51]. These discrepancies may be explained by the use of different OPC markers and/or distinctions in the time frame of oligodendroglial response between white and gray matter structures [45,47]. The results of our study indicate close quantitative agreement between molecular and cellular components of myelin loss and repair in the cuprizone model including myelin content changes assessed by both MBP and MPF, and the number of myelinating oligodendrocytes and OPC in all anatomic structures. While this study was not focused on the elucidation of detailed temporal profiles of cellular responses during de- and remyelination, our data suggest a high degree of synchrony between underlying cellular events in both white and gray matter in agreement with earlier studies [44,46].

The presented results have several implications for the design of future preclinical studies of remyelinating therapies. First, based on very strong correlations between MPF and histological myelin content measures, this and previous [17,18,19] studies suggest that fast MPF mapping can be used interchangeably with myelin histology in animal studies, particularly if several time points are needed. Our results demonstrate that the effect sizes for MPF, while being slightly lower than those for histological variables, are still very large (>2 for all anatomic structures). Additional gain in statistical power can be achieved due to longitudinal measurements using MPF as opposed to the study designs based on histological endpoints only. Second, this study shows that both imaging and histological outcomes in the cuprizone model should be assessed not only for the corpus callosum but also for different gray matter structures (cortex, striatum, hippocampus), where the effects of both demyelination and remyelination are highly significant. Cortical demyelination is known to represent a separate and highly clinically relevant aspect of MS pathology [10,11,12], while cortical MS lesions have been shown to hold an extensive remyelination potential compared to white matter lesions [52,53]. Accordingly, the advent of an imaging method enabling reliable assessment of cortical remyelination may substantially impact future preclinical and clinical studies of myelin repair therapies. Third, our results may be helpful in the interpretation of the OPC count as an outcome measure in preclinical myelin repair studies. The dynamics of OPC population is known to demonstrate a biphasic behavior with an increase during cuprizone-induced demyelination followed by a decrease during recovery after cuprizone discontinuation [22,23,24,25,26]. However, the significance of OPC count as a biomarker in preclinical studies of remyelination therapies using the cuprizone model remains an open question due to a possible mismatch between temporal profiles of mature oligodendrocyte repopulation, remyelination, and OPC proliferation. Recent meta-analysis [5] showed that the histological or immunohistochemical myelin assessment and oligodendrocyte count provided consistent results as outcome measures for prospective remyelination therapies, while the performance of OPC count was rather controversial with a non-significant overall effect. Our results demonstrate strong negative correlations between the OPC count and both the population of myelinating oligodendrocytes and myelin content measures (MBP and MPF). These correlations indicate that OPC count may provide extra confidence in the assessment of therapeutic efficacy and suggest that its reduction towards the normal level should be viewed as a favorable treatment outcome in acute demyelination settings. At the same time, more research is needed to identify the utility of OPC assessment in the treatment of chronic demyelination where the OPC pool may be intrinsically depleted [54].

While this study provides a compelling evidence of the utility of fast MPF mapping as a means to quantify remyelination in the acute cuprizone intoxication model, some aspects of the application of this method to other animal models of MS need further research. Besides cuprizone, common MS models include inflammatory demyelination caused by immunization with myelin antigens (experimental autoimmune encephalomyelitis (EAE)) or infectious agents (Theiler’s murine encephalomyelitis virus and murine hepatitis virus) and toxic demyelination induced by focal administration of lysolecithin or ethidium bromide [25,55]. Newer models involving different demyelination mechanisms, such as inducible conditional knock-out of the myelin regulatory factor (iCKO-Myrf) in mice [56] and feline irradiated diet-induced demyelination (FIDID) [57] were also described. All these models capture certain pathological features of MS, though there is no single model that could entirely mimic the human disease. In contrast to acute cuprizone intoxication where remyelination is typically rapid and complete [22,23,24,25,26], only partial remyelination occurs in some other models including prolonged cuprizone administration [22,23,24,25,26,54], EAE [25,55], FIDID [57], and iCKO-Myrf [56]. Arguably, these models may be more relevant to chronic MS lesions in humans, where complete remyelination usually does not happen [1,6,7]. Due to a reduced effect size associated with remyelination, such models may pose challenges in the application of MPF mapping or any other quantitative imaging approach for the assessment of remyelination in either spontaneous or treatment-related settings. Another potential challenge may be associated with edema, which is a common feature of the inflammatory models [58] and present to a lesser extent in the cuprizone model [59]. A recent study [18] demonstrated that MPF measurements in the acute ischemic stroke model may be confounded by edema, though myelin remains the main factor driving MPF changes in the ischemic lesion. More sophisticated MPF mapping techniques with a reduced sensitivity to tissue water content alterations [60,61] may be advantageous in the applications to inflammatory MS models as well as MS lesions in humans.

The key practical advantage of the fast MPF mapping method is the simplicity of clinical translation. This method has already been successfully applied in several clinical studies of MS [12,15] and traumatic brain injury [16] with the use of 3 Tesla research MRI equipment. In these studies [12,15,16], MPF mapping demonstrated the capability to quantify demyelination not only in white matter but also in cortical and subcortical gray matter. Recent studies [19,20,62] demonstrated the feasibility of MPF mapping using a routine 1.5 Tesla clinical MRI scanner in conjunction with the design of ultrafast protocols enabling collection of source images within a few minutes. Furthermore, due to the inherent insensitivity of MPF to magnetic field strength [63], the data obtained in humans on clinical scanners can be quantitatively compared to the values measured in animal models with the use of specialized high-field MRI equipment. An additional advantage of MPF mapping in both clinical and preclinical studies is its independence of changes in tissue relaxation times T_1_ and T_2_ caused by paramagnetic ions, particularly iron [15]. Abnormal iron deposition in the subcortical gray matter anatomic structures is a known pathological feature of MS [15,64,65]. A growing body of evidence suggests that an altered iron metabolism is closely associated with myelin pathology in both human MS disease and animal demyelination models [66,67,68,69]. Sensitivity to tissue relaxation properties is a known problem in alternative approaches for myelin imaging, such as multi-component relaxation methods [70,71,72] and MTR, while MPF mapping overcomes this limitation [12,15]. Taken together, the results of this and previous applications [12,13,14,15,16,17,18,19,20,21] suggest that the fast MPF mapping technology can provide a method of choice for preclinical and clinical studies of myelin repair therapies.

## 5. Conclusions

The results of this study provide comprehensive immunohistological validation of the fast MPF mapping method as a non-invasive quantitative tool for preclinical and clinical studies of de- and remyelination in both white and gray matter. Strong correlations between MPF and quantitative immunochemistry of the major protein component of myelin (MBP) support the use of MPF as a myelin biomarker in a variety of neurological conditions. Our results also demonstrate close quantitative agreement between oligodendroglial response and myelin content changes in the cuprizone model. Correlations of MPF with both myelin content and oligodendrogenesis indicate the feasibility of using this parameter as a uniform surrogate marker of reparative processes in demyelinating diseases.

## Figures and Tables

**Table 1:** Effect sizes (Cohen’s d) with 95% confidence intervals (CI) corresponding to the demyelination and remyelination states in the corpus callosum, caudate putamen, cortex, and hippocampus based on MPF and quantitative histology variables.

Variable	Control-Demyelination: d [−CI, +CI]				Demyelination-Remyelination: d [−CI, +CI]			
	CC	CPu	Cor	Hip	CC	CPu	Cor	Hip
MPF	2.7 [0.8, 4.6]	2.3 [0.5, 4.5]	2.2 [0.5, 4.0]	2.2 [0.5, 4.0]	−2.3 [−4.0, −0.6]	−2.1 [−3.7, −0.5]	−1.8 [−3.4, −0.3]	−2.8 [−4.7, −1.0]
MBP area percentage	3.9 [1.6, 6.3]	3.8 [1.5, 6.1]	5.6 [2.2, 8.0]	4.6 [1.9, 7.2]	−3.1 [−5.1, −1.2]	−2.8 [−4.6, −0.9]	−4.4 [−6.8, −2.0]	−4.0 [−6.3, −1.7]
CNP-positive cell count	5.7 [2.6, 8.8]	3.8 [1.5, 6.1]	2.9 [0.9, 4.9]	3.0 [1.9, 7.2]	−5.4 [−8.2, −2.6]	−4.3 [−6.6, −1.9]	−3.5 [−5.6, −1.4]	−2.4 [−4.2, −0.7]
NG2-positive cell count	−4.4 [−7.0, −1.8]	−1.7 [−7.0, −1.8]	−3.5 [−7.0, −1.8]	−2.6 [−7.0, −1.8]	2.7 [0.9, 4.6]	1.6 [0.1, 3.2]	4.0 [1.7, 6.2]	3.0 [1.1, 4.9]

**Table 2:** Linear regression analysis of associations between MPF, MBP-stained percentage of the total area, CNP-positive cell count, and NG2-positive cell count in the corpus callosum, caudate putamen, hippocampus, and cortex.

Parameters	Brain Structure	r	r^2^	*p*	Slope (95% CI), *p*	Intercept (95% CI), *p*
MPF vs. MBP % total area	CC	0.90	0.82	<0.001	0.06 (0.04, 0.07), <0.001	7.84 (6.57, 9.12), <0.001
	CPu	0.83	0.69	<0.001	0.04 (0.02, 0.06), <0.001	6.57 (5.68, 7.46), <0.001
	Hip	0.80	0.64	< 0.001	0.06 (0.03, 0.09), 0.001	6.20 (5.46, 6.95), <0.001
	Cor	0.88	0.78	<0.001	0.04 (0.02, 0.05), <0.001	7.04 (6.52, 7.56), <0.001
MPF vs CNP + cells	CC	0.84	0.71	<0.001	0.57 (0.33, 0.81), <0.001	8.96 (7.70, 10.21), <0.001
	CPu	0.71	0.51	0.006	0.38 (0.13, 0.63), 0.006	6.87 (5. 76, 7.98), <0.001
	Hip	0.70	0.50	0.007	0.77 (0.26, 1.29), 0.007	5.78 (4. 54, 7.01), <0.001
	Cor	0.79	0.63	0.001	0.35 (0.17, 0.53), 0.001	6.49 (5.46, 7.51), <0.001
MPF vs NG2 + cells	CC	−0.69	0.47	0.009	−0.82 (−1.40, −0.25), 0.009	14.83 (12.57, 17.10), <0.001
	CPu	−0.76	0.58	0.006	−0.67 (−1.04, −0.29), 0.002	14.67 (12.94, 16.41), <0.001
	Hip	−0.75	0.57	0.003	−0.60 (−0.95, −0.25), 0.003	8.79 (7.95, 9.63), <0.001
	Cor	−0.77	0.59	0.002	−0.34 (−0.52, −0.15), 0.002	10.43 (9.28, 11.59), <0.001
MBP % total area vs CNP + cells	CC	0.92	0.85	<0.001	10.13 (7.36, 12.87), <0.001	20.32 (6.02, 34.61), 0.01
	CPu	0.85	0.72	<0.001	8.49 (4.46, 12.52), <0.001	7.47 (−9.04, 23.98), 0.34
	Hip	0.82	0.67	<0.001	11.43 (6.11, 16.74), <0.001	−4.90 (−17.57, 7.77), 0.41
	Cor	0.81	0.66	<0.001	8.49 (4.46, 12.52), <0.001	−10.05 (−32.95, 12.86), 0.36
MBP % total area vs NG2 + cells	CC	−0.72	0.62	0.005	−13.87 (-22.78, −4.96), 0.005	121.88 (86.93, 156.82), <0.001
	CPu	−0.81	0.65	<0.001	−8.21 (−12.18, −4.25), <0.001	81.81 (63.41, 100.20), <0.001
	Hip	−0.74	0.54	0.004	−7.45 (−11.98, −2.91), 0.004	36.59 (25.57, 47.62), <0.001
	Cor	−0.89	0.78	<0.001	−9.14 (−12.32, −5.96), <0.001	91.48 (71.83, 111.13), <0.001
CNP + cells vs NG2 + cells	CC	−0.79	0.62	0.001	−1.39 (−2.11, −0.67), 0.001	10.10 (7.28, 12.93), <0.001
	CPu	−0.68	0.47	0.01	−0.66 (−1.12, −0.19), 0.01	7.14 (4.99, 9.31), <0.001
	Hip	−0.79	0.62	0.001	−0.57 (−0.87, −0.27), 0.001	3.45 (2.73, 4.17), <0.001
	Cor	−0.63	0.39	0.02	−0.62 (−1.13, −0.11), 0.02	9.22 (6.07, 12.38), <0.001

Abbreviation: CI, confidence interval; CC, corpus callosum; CPu, caudate putamen; Hip, hippocampus; Cor, cortex.
